# Prolapsus Uteri during Parturition

**Published:** 1850-03

**Authors:** 


### OBSTETRICS.

*Prolapsus Uteri during Parturition.* By Dr. Harting.—Called by a midwife to a primipara, Dr. Harting was astonished at finding that, under the influence of strong and continuous pain, the lower half of the pregnant uterus had become propelled beyond the external parts. The os uteri was closed, and so rigid, that the point of the finger was forced through it only with great difficulty. The membranes were not yet ruptured, and the child's knee was felt through them. All attempts at returning the organ only excited great suffering, and induced a bearing down that aggravated the evil. A crucial incision was made into the indurated os, the liquor amnii discharged, the child's knee seized, and delivery gradually accomplished, the midwife supporting the uterus the while. After the removal of the placenta, the uterus was easily returned, and the patient, after having been kept on her back for three weeks, did not find the prolapsus reappear on resuming the erect posture.—*Brit. & For. Med. Chir. Rev. from Medicinische Zietung.*

